# Neovascular age‐related macular degeneration without drusen

**DOI:** 10.1111/aos.70010

**Published:** 2025-10-15

**Authors:** Marc J. Sirks, Elon H. C. van Dijk, Husein Ghalayini, Somayeh Bazdar, WeiFeng Yu, Suzanne Yzer, José P. Martinez Ciriano, Reinier O. Schlingemann, Roselie M. H. Diederen, Camiel J. F. Boon

**Affiliations:** ^1^ Department of Ophthalmology Amsterdam University Medical Center Amsterdam The Netherlands; ^2^ Department of Ophthalmology Leiden University Medical Center Leiden The Netherlands; ^3^ Rotterdam Eye Hospital Rotterdam The Netherlands; ^4^ Department of Ophthalmology Radboud University Medical Center Nijmegen The Netherlands; ^5^ Department of Ophthalmology, Ocular Angiogenesis Group Amsterdam University Medical Center Amsterdam The Netherlands; ^6^ Department of Ophthalmology Jules‐Gonin Eye Hospital, Fondation Asile Des Aveugles Lausanne Switzerland

**Keywords:** age‐related macular degeneration, anti‐VEGF, drusen, macular neovascularization, photodynamic therapy, polypoidal choroidal vasculopathy

## Abstract

**Purpose:**

To describe the clinical characteristics and 1‐year follow‐up of patients with neovascular AMD (nAMD) without drusen in either eye.

**Methods:**

This is a multicentre retrospective cohort study in three tertiary referral centres in The Netherlands. We included patients of 55 years or older with nAMD in one or both eyes, without the presence of drusen or signs of another underlying disease in either eye. The medical charts and multimodal imaging (MMI) were evaluated by two independent graders. Eyes were divided into two groups: polypoidal choroidal vasculopathy (PCV)‐associated macular neovascularization (MNV) and non‐PCV MNV. We evaluated the visual acuity (VA) at baseline and 1 year after baseline; complete resolution of macular fluid on optical coherence tomography (OCT) during 1 year of follow‐up; required treatments to achieve a complete resolution of macular fluid on OCT; complication rate.

**Results:**

We included 106 eyes of 99 patients, with a median age of 73 years. Seventy‐one eyes had PCV‐associated MNV, and 35 eyes had non‐PCV MNV. The overall median baseline VA was 0.22 logMAR (Snellen 20/30), and 0.15 logMAR (Snellen 20/28) at 1 year follow‐up. Subretinal haemorrhage was more common in PCV‐associated MNV compared to non‐PCV MNV, both at initial presentation (21% vs. 17%) and during the 1 year follow‐up period (23% vs. 11%). In total, 31 out of 48 eyes (65%) achieved complete resolution of macular fluid on OCT during follow‐up, most commonly achieved with combined photodynamic therapy (PDT) + intravitreal anti‐vascular endothelial growth factor (anti‐VEGF) injections.

**Conclusion:**

nAMD can occur without the presence of drusen in either eye. Treatment with anti‐VEGF injections, PDT or a combination thereof appears to be effective, but more research is warranted.

## 1 INTRODUCTION

Age‐related macular degeneration (AMD) is a disease that affects the retina and underlying choroid. Its prevalence lies at 8.7–11.6% of the global population and increases with age (Rein et al., 2022; Wong et al., 2014). It occurs in people over 55 years of age (Guymer & Campbell, 2023), and a recent meta‐analysis has found a pooled prevalence as high as 25.3% in Europeans over 60 years old (Li et al., 2020). With an increase in longevity and an increasing aging population, the prevalence of AMD is expected to rise over the coming decades (Wong et al., 2014), while it is already the fourth most frequent cause of blindness worldwide and the second most frequent cause of blindness in the Western world (GBD 2019 Blindness and Vision Impairment Collaborators, 2021). AMD can be divided into several stages: early, intermediate and late AMD (National Institute for Health and Care Excellence, 2018). Early AMD is characterized by medium drusen (larger than 63 μm but smaller than 125 μm) or large drusen (larger than 125 μm) or any AMD‐related pigmentary changes. There are two main forms of late AMD: geographic atrophy or neovascular AMD (nAMD). The progression of the disease is generally described as a path from small drusen to medium drusen and/or more extensive pigmentary changes to large drusen and finally to late AMD in the form of geographic atrophy and/or macular neovascularization (MNV) (Vujosevic et al., 2023).

Drusen are an accumulation of materials in the extracellular space between the basal lamina of the retinal pigment epithelium (RPE) and Bruch's membrane. Drusen are composed of different biomaterials, including lipids, complement proteins and many other proteins, and their composition may differ between eyes or even within eyes (Bergen et al., 2019; Curcio, 2018). Some of these components are secreted only by RPE cells or photoreceptors, some are only found in the blood serum, and others find their origin both in local tissues and the circulation (Bergen et al., 2019). The increase in drusen number and size can cause hypoxia at the apex of the RPE detachment inducing apoptosis, resulting in outer retinal atrophy (Curcio, 2018). MNV most commonly originates from the choroidal vasculature and has been postulated to occur in AMD as a result of factors such as oxidative stress, ischemia and senescence in a pro‐inflammatory environment of drusen (Schlingemann, 2004; Spaide et al., 2003).

We have previously shown in a cohort of 29 patients that there may be a clinical picture of nAMD in which MNV is present without accompanying drusen in the affected or in the fellow eyes unaffected by MNV, and without signs of any other underlying diagnosis that may cause MNV (Chung et al., 2017). It is not yet known what the underlying cause is of such cases of MNV in ‘nAMD without drusen’, and whether or not this phenotype shows pathophysiologic overlap with drusen‐associated nAMD or other types of MNV. In addition, the clinical course and response to treatment of this disease entity have not been extensively described to date. In this study, we present the clinical characteristics, follow‐up and treatment outcome in a large cohort of patients with nAMD without drusen in either eye.

## 2 METHODS

### 2.1 Study population

This is a retrospective study describing patients with nAMD without the presence of drusen in either eye. Patients who visited one of three tertiary referral centres in The Netherlands (Amsterdam University Medical Center, Leiden University Medical Center and Rotterdam Eye Hospital) and who were suspected of having nAMD without drusen were assessed for eligibility. Initial visits to the aforementioned centres were between 1 January 2009 and 1 January 2025. We used the following inclusion criteria: age of 55 years or older at initial presentation with an MNV; multimodal imaging (MMI), including optical coherence tomography (OCT), OCT‐angiography (OCT‐A), colour fundus photography (CFP), fundus fluorescence angiography (FFA) or indocyanine green angiography (ICGA), of sufficient quality to confirm the presence of an MNV in one or both eyes; MMI of sufficient quality to rule out the presence of any medium drusen (between 63 and 125 μm in diameter) or any large drusen (larger than 125 μm in diameter) in the macular area and the periphery of both eyes. In addition, we wanted to exclude patients with extensive small drusen (smaller than 63 μm), by only including patients with less than five small drusen in the affected or fellow eye. Exclusion criteria were as follows: myopia of more than six dioptres; previous retinal surgery; presence of other vascular retinal diseases or diseases causing MNVs, such as diabetic macular oedema or retinal vascular occlusions; presence of five or more small drusen or any medium or large drusen on any future MMI that was available up until January 2025; presence of diseases that are part of the pachychoroid disease spectrum, which was defined as the presence of (multi)focal indistinct signs of choroidal hyperpermeability (FISH) or diffuse indistinct signs of choroidal hyperpermeability (DISH; Pauleikhoff et al., 2024), and may be accompanied by the presence of pachyvessels on OCT or ICGA (Cheung et al., 2024). If MNV was present bilaterally, sufficient MMI was required to be available from either eye before the development of the MNV, to rule out the presence of drusen. This study adhered to the tenets of the Declaration of Helsinki, and the local institutional review board ruled that approval was not required for this study. Informed consent was obtained from all included patients.

Retrospective analysis of the following MMI modalities was performed: OCT, OCT‐A, CFP, FFA and ICGA. Because of the retrospective multicentre setting of the current study and the long inclusion period, many different imaging devices were used. The OCT scans were made with devices manufactured by: Spectralis HRA + OCT, Heidelberg Engineering GmbH (Heidelberg, Germany), Topcon Corp. (Tokyo, Japan), Carl Zeiss Meditec (Dublin, CA, USA) or Canon (Tokyo, Japan). OCT‐A scans were made with devices manufactured by Optovue Inc. (Fremont, CA, USA) and Canon. The colour fundus pictures were made with devices manufactured by Topcon Corp. and Optos (Dunfermline, UK). FFA and ICGA images were made with devices manufactured by Spectralis HRA + OCT, Heidelberg Engineering GmbH, Topcon Corp. and Optos. If both an ICGA performed on a digital flash camera system and a confocal scanning laser ophthalmoscopy (SLO) system were available, imaging analysis was performed on the confocal SLO‐based ICGA. All MMIs that were available 4 months prior to or after the baseline visit were included in the analysis for the baseline visit. For the 1 year follow‐up visit, a visit occurring between 8 and 16 months after the baseline visit was considered eligible for this study. All MMIs within this timeframe were included in the analysis for the 1 year follow‐up visit.

### 2.2 Clinical assessment

The abovementioned MMI was assessed by two independent graders, and any dissensus between these graders was resolved by a referee. For all cases, MJS was the first grader, and HG or SB was the second grader. The referees were CJFB, RMHD and EHCvD. Any discrepancies in measurements of continuous variables (e.g. central retinal thickness) were averaged if the difference was less than 10%. If the difference was greater than 10%, a re‐evaluation of the measurement took place, or it was discussed with one or more referees. The external limiting membrane, ellipsoid zone and RPE integrity were graded as continuous, irregular/thinned or indiscernible, based on available OCT scans. In the presence of foveal subretinal fluid, the ellipsoid zone integrity was still scored unless there was too much uncertainty to allow accurate scoring, in which case the ellipsoid zone integrity was considered missing. A grading manual of all assessed parameters was used to ensure consistent grading between independent graders and referees over time.

All VA measurements were changed from Snellen decimal notation to logMAR units using the following formula: logMAR VA = −log(decimal VA).

### 2.3 Statistical analyses

All statistical analyses were performed in IBM SPSS Statistics, version 28 (IBM, Armonk, NY, USA). For normally distributed continuous data, mean and standard deviation were used. For non‐normally distributed continuous data, median and interquartile range (IQR) were used. For comparative analyses between groups, the Mann–Whitney *U* test was used for continuous data, and the chi‐square test was used for dichotomous data. A Bonferroni‐adjusted *p*‐value of lower than 0.00217 was considered statistically significant.

## 3 RESULTS

### 3.1 Patient characteristics and referral patterns

**FIGURE 1:**
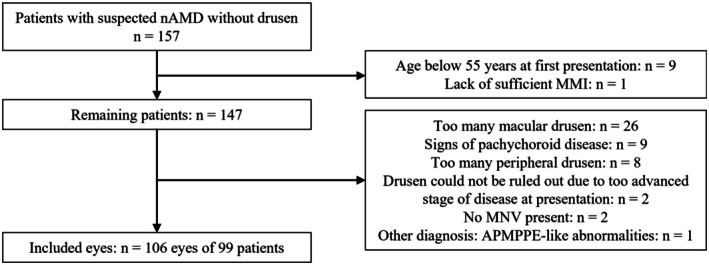
Flowchart of application of inclusion and exclusion criteria. APMPPE, acute posterior multifocal placoid pigment epitheliopathy; MMI, multimodal imaging; *n*, number; nAMD, neovascular age‐related macular degeneration.

**FIGURE 2:**
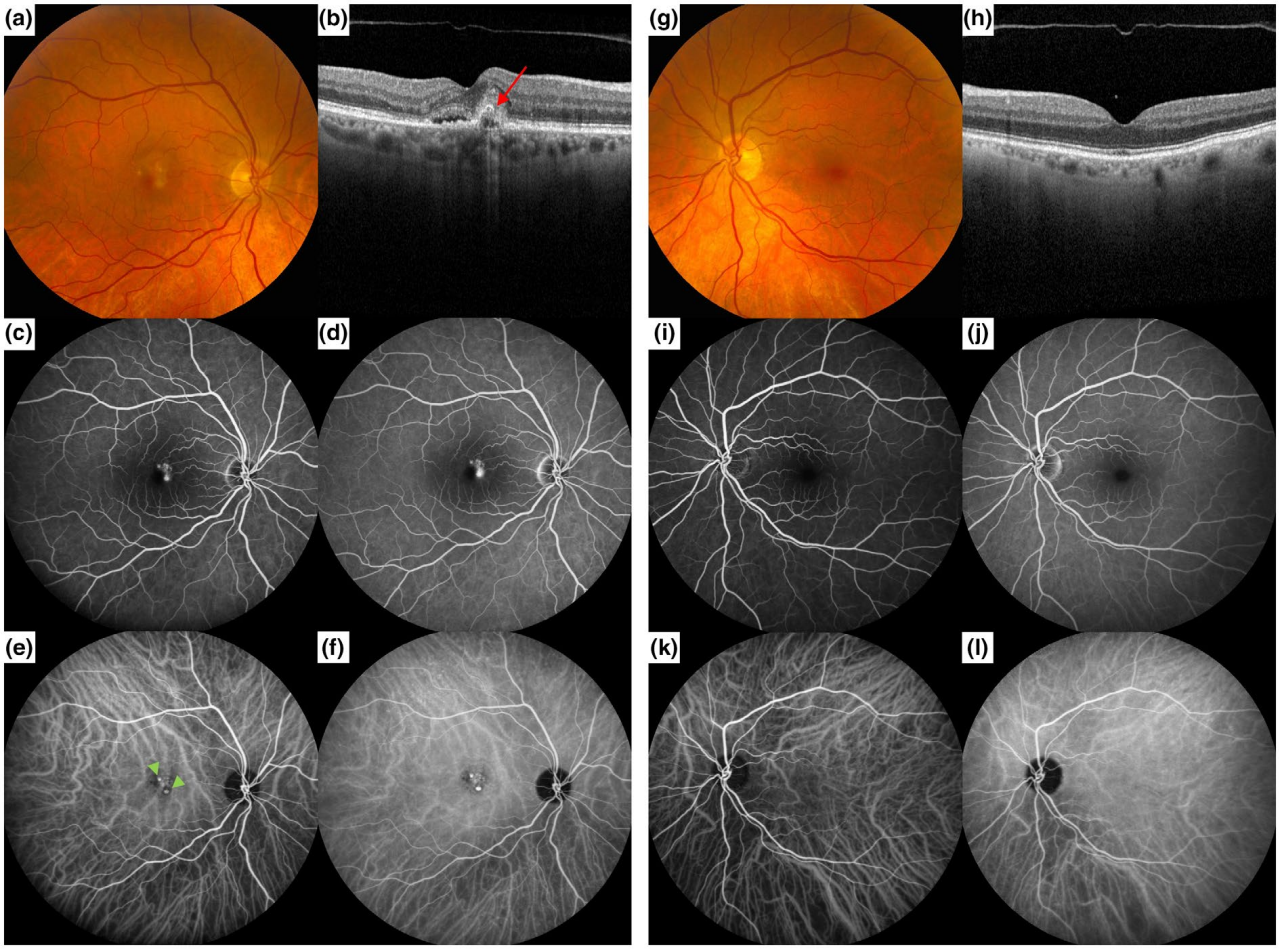
Example case of a 67‐year‐old woman with neovascular age‐related macular degeneration (AMD) and an associated PCV component, without drusen. (a) Colour fundus photograph (CFP) of the eye affected by macular neovascularization (MNV), showing RPE alterations in the macular area, but no drusen; (b) Foveal optical coherence tomography (OCT) showing subretinal fluid, a flat irregular pigment epithelium detachment with a polypoidal lesion (PL; red arrow) at the edge of the lesion. The choroidal thickness is normal. (c) Early phase fundus fluorescein angiography (FFA) showing the hyperfluorescent MNV; with mid‐ to late phase FFA (d) showing leakage; (e) Early phase indocyanine green angiography (ICGA) shows two PLs in the macular area; (f) Mid‐phase ICGA still showing the two PLs in the macular area, but no focal or diffuse indistinct signs of choroidal hyperpermeability or other underlying disease (Pauleikhoff et al., 2024); (g) CFP of the fellow eye, showing no drusen or retinal pigment epithelium alterations; (h) Normal foveal OCT scan with a normal choroidal thickness; FFA (i, j) and ICGA (k, l) have a normal aspect.

**FIGURE 3:**
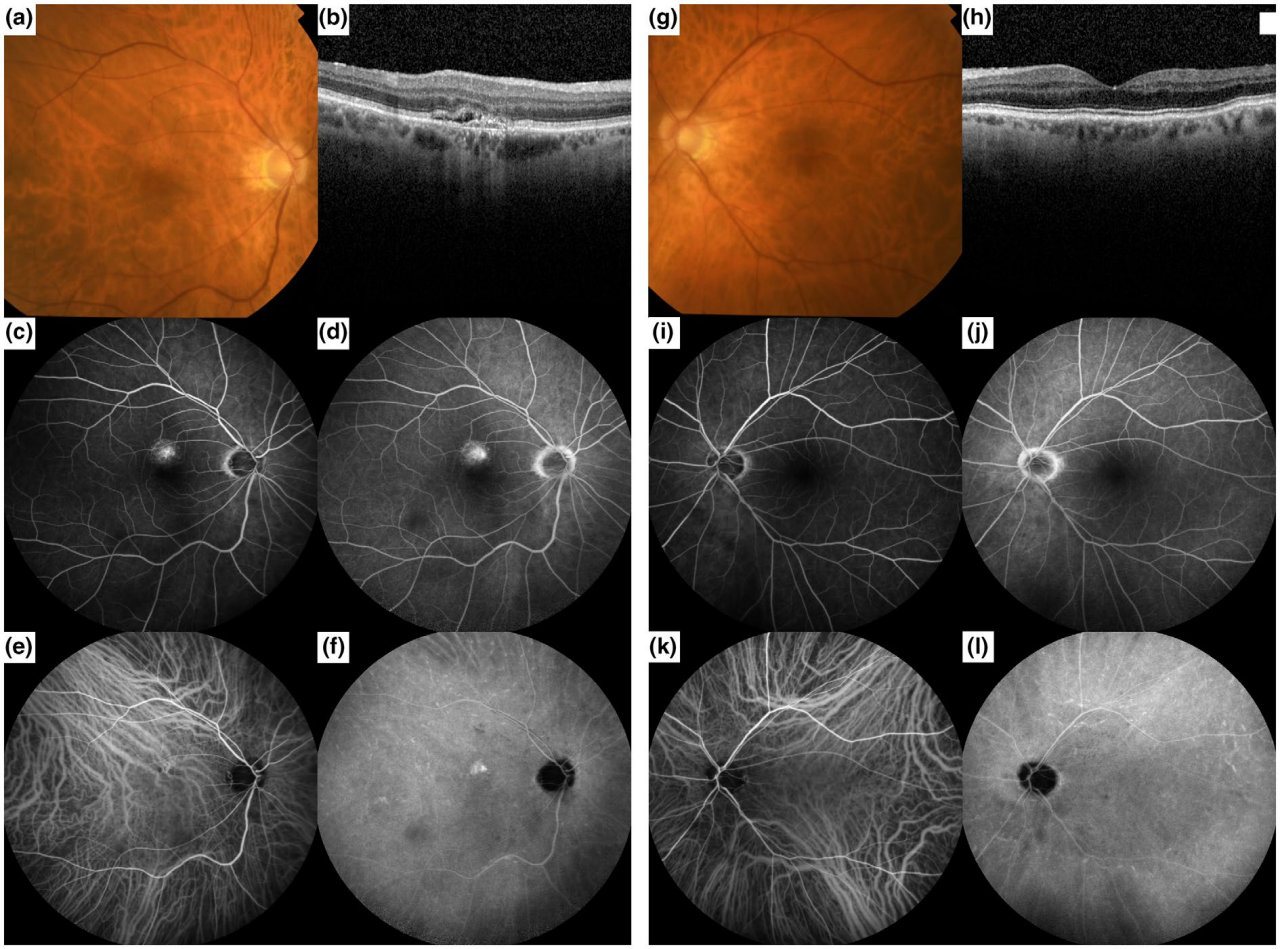
Example case of a 70‐year‐old man with neovascular age‐related macular degeneration (AMD) without drusen. (a) Colour fundus photograph (CFP) showing subtle RPE alterations just superior to the fovea, but no drusen; (b) Optical coherence tomography (OCT) scan showing subretinal fluid and a flat irregular pigment epithelium detachment with the underlying type 1 macular neovascularization (MNV). The choroidal thickness is within the normal range. (c) Early phase fundus fluorescein angiography (FFA) showing hyperfluorescence just superior to the fovea; (d) Mid‐phase FFA shows the macular hyperfluorescent leakage; (e) Early phase indocyanine green angiography (ICGA) showing the presence of an MNV just superior to the fovea, but no polypoidal lesions; (f) Mid‐phase ICGA showing mild and well‐demarcated hyperfluorescence superior to the fovea, but no focal or diffuse indistinct signs of choroidal hyperpermeability (Pauleikhoff et al., 2024); (g) CFP of the fellow eye, showing no drusen or retinal pigment epithelium alterations; (h) Normal foveal OCT scan with a normal choroidal thickness; FFA (i, j) and ICGA (k, l) also had a normal aspect.

An initial 157 patients with suspected nAMD without drusen were evaluated for this study. After applying the strict in‐ and exclusion criteria, 106 eyes of 99 patients were included (see Figure 1). Out of all MNVs found, 71 had the presence of (a component of) PCV (67%). The most commonly found MNV was type 1 MNV in combination with a PCV component (type B PCV (van Dijk et al., 2021); 51% of eyes), followed by type 1 MNV without PCV (28% of eyes), isolated PCV (without an associated flat type 1 neovascular membrane); type C PCV (van Dijk et al., 2021; 12% of eyes), mixed type MNV with PCV (4% of eyes), followed by less frequently occurring MNVs without PCV such as type 2 MNV (2.8%) and mixed type MNV (1.9%; van Dijk et al., 2021). Three eyes with a type 1 MNV without PCV developed PCV after 1.5, 2.5 and 7 years, respectively. There was one patient with a type 1 MNV without PCV in 1 eye and a type 1 MNV with PCV in the other eye at baseline. Example cases of patients with nAMD without drusen are displayed in Figures 2 and 3.

The median age at presentation was 73 years (interquartile range: 66–79 years old), and 52% of patients were male. All patients were of Caucasian ethnicity. Bilateral MNVs were found in 7 out of 99 patients (7%). Five out of these seven patients presented with bilateral MNV at the same visit. In the other two cases, an MNV was discovered 3 and 6 years later, respectively. Information about family history for AMD was available for 36 patients (36%). Six out of 36 patients had a positive family history for AMD (17%), out of whom four had a single affected family member (a parent in three occasions, a sibling in one occasion), one patient had three affected family members, and one patient had at least 5 affected family members (all maternally related). The latter patient received extensive genetic testing, but no potentially disease‐associated genetic risk factors were identified. Information on smoking history was available for 29 patients (29%). Fourteen of these 29 patients were either current or past smokers (48%). The mean spherical refraction of the affected eyes that had not had cataract surgery was +1.39 dioptres, with a standard deviation of 2.68 dioptres.

Of all 99 patients, 9 were already under the care of an ophthalmologist at 1 of our centres, and the remaining 90 were referred to us at the moment of inclusion. The referrals were made by an ophthalmologist in 75 cases (83% of referrals), by a general physician in 13 cases (14% of referrals) and by an optometrist in 2 cases (2% of referrals). In referrals from ophthalmologists, the following reasons for referral were mentioned: suspicion of an alternative diagnosis than AMD (68%), assessment of a potential indication to perform PDT (28%), unsatisfactory response to anti‐vascular endothelial growth factor (VEGF) treatment (27%), to perform ICGA (25%), to evaluate a potential indication for focal laser treatment (4%) or a previous episode of PCV (4%). At the baseline visit, 57% of eyes were treatment naïve, 33% had received intravitreal anti‐VEGF injections, 8% of eyes had received focal laser treatment, 4% had received non‐steroidal anti‐inflammatory drug eye drops, 3% of eyes had received PDT and 1% had received corticosteroid eye drops. Of those eyes that had received intravitreal anti‐VEGF injections prior to referral, 62% had received one type of anti‐VEGF treatment, 18% had received two types of anti‐VEGF treatment and 21% had received three types of anti‐VEGF treatment. These patients had received between 2 and 33 anti‐VEGF injections before the baseline moment of this study, with a median of 6 injections (interquartile range: 3–16).

### 3.2 Baseline characteristics of eyes with nAMD without drusen

At baseline, the following MMI modalities were available: OCT in 103 eyes (97%), CFP in 90 eyes (85%), FFA in 96 eyes (91%) and ICGA in 89 eyes (84%). For 48 out of 89 eyes (54%), a confocal SLO‐based ICGA was available, and for the remaining 41 out of 89 eyes (46%), a digital flash camera‐based ICGA was used. Baseline characteristics can be found in Table 1.

**TABLE 1:** Baseline characteristics.

	PCV‐associated MNV (71 eyes of 68 patients)	Non‐PCV MNV (35 eyes of 32 patients)	*p*‐value	Total (106 eyes of 99 patients)^c^
Visual acuity in logMAR [IQR]—affected eye	0.15 [0.10–0.40]	0.22 [0.05–0.40]	0.766^a^	0.22 [0.05–0.40]
Visual acuity in logMAR [IQR]—fellow eye	0 [0–0.10]	0 [−0.08–0.05]	0.122^a^	0 [−0.08–0.10]
CFP characteristics				
Subretinal haemorrhage	14/68 (21%)	6/35 (17%)	0.675^b^	20/103 (19%)
Area of subretinal haemorrhage				
<1× disc area	10/14 (71%)	3/6 (50%)		12/19 (63%)
1–3 disc areas	2/14 (14%)	3/6 (50%)		5/19 (26%)
>3 disc areas	2/14 (14%)	N/A		2/19 (11%)
Hard exudates	40/68 (59%)	6/35 (17%)	<0.001^b^	46/103 (45%)
Pigmentary changes	47/66 (71%)	30/34 (88%)	0.055^b^	77/100 (77%)
OCT characteristics				
Subretinal fluid	62/68 (91%)	28/33 (85%)	0.338^b^	90/101 (89%)
Subfoveal	38/62 (61%)	22/28 (79%)		60/90 (67%)
Intraretinal fluid	23/65 (35%)	8/33 (24%)	0.262^b^	31/98 (32%)
Foveal	11/23 (48%)	7/8 (88%)		18/31 (58%)
Hyperreflective foci	46/67 (69%)	16/33 (48%)	0.051^b^	62/100 (62%)
Subretinal hyperreflective material	20/60 (33%)	10/33 (30%)		30/93 (32%)
Foveal subretinal hyperreflective material	6/20 (30%)	7/10 (70%)		13/30 (43%)
Foveal RPE detachment	24/70 (34%)	22/32 (69%)	0.001^b^	46/102 (45%)
MNV area in mm^2^	3.31 [1.51–7.73]	1.60 [0.77–6.88]	0.141^a^	2.54 [1.02–7.42]
MNV involving macula	68/71 (96%)	35/35 (100%)		103/106 (97%)
Involving fovea	24/68 (35%)	23/35 (66%)		47/103 (46%)
Foveal retinal thickness	131 [107–144]	114 [98–156]	0.656^a^	128 [104–150]
Subfoveal choroidal thickness	238 [199–283]	197 [164–244]	0.038^a^	225 [181–269]
FIPED	48/64 (75%)	19/29 (66%)	0.345^b^	67/93 (72%)
Greatest FIPED width	1758 [1374–2453]	1220 [878–1835]		1580 [1167–2372]
Greatest width of total RPE detachment	1933 [1344–2570]	1225 [867–2708]		1819 [1084–2570]
Subfoveal ELM integrity				
Continuous	39/55 (71%)	16/22 (73%)		55/77 (71%)
Irregular/thinned	14/55 (25%)	4/22 (18%)		18/77 (23%)
Indiscernible	2/55 (4%)	2/22 (9%)		4/77 (5%)
Subfoveal EZ integrity				
Continuous	24/55 (44%)	/22 (18%)		28/77 (36%)
Irregular/thinned	22/55 (40%)	15/22 (68%)		37/77 (48%)
Indiscernible	9/55 (16%)	3/22 (14%)		12/77 (16%)
Subfoveal RPE integrity				
Continuous	36/55 (65%)	15/23 (65%)		51/78 (65%)
Irregular/thinned	19/55 (35%)	7/23 (30%)		26/78 (33%)
Indiscernible	N/A	1/23 (4%)		1/78 (1%)
FFA‐based parameters				
Leakage on FFA	49/61 (80%)	30/35 (86%)	0.506^b^	79/96 (82%)
Focal leakage	37/49 (76%)	20/30 (67%)		57/79 (72%)
Diffuse leakage	12/49 (24%)	10/30 (33%)		22/79 (28%)
Early leakage	40/46 (87%)	27/30 (90%)		67/76 (88%)
Late leakage	9/46 (20%)	13/30 (43%)		22/76 (29%)

Abbreviations: CFP, colour fundus photography; ELM, external limiting membrane; EZ, ellipsoid zone; FFA, fundus fluorescein angiography; FIPED, flat irregular pigment epithelium detachment; IQR, interquartile range; logMAR, logarithm of the minimal angle of resolution; MNV, macular neovascularization; N/A, not applicable; OCT, optical coherence tomography; PCV, polypoidal choroidal vasculopathy; RPE, retinal pigment epithelium.

^a^ Mann–Whitney *U* test.

^b^ Chi‐squared test.

^c^ There was one patient who had one eye with a PCV‐associated MNV and 1 eye with a non‐PCV MNV.

### 3.3 One year follow‐up

One year follow‐up was available for 57 out of 106 eyes (54%). The following MMI modalities were available at 1 year follow‐up: OCT in all 57 eyes, CFP in 51 out of 57 eyes (89%), FFA in 6 out of 57 eyes (11%) and ICGA in 13 out of 57 eyes (23%). For 5 out of 13 eyes (38%), a confocal SLO‐based ICGA was available and for the remaining 8 out of 13 eyes (62%), a digital flash camera‐based ICGA was used. The received treatments during the 1 year follow‐up period are listed in Table 2. In 22 out of 32 eyes (69%) with PCV‐associated MNV, a complete resolution of intra‐ or subretinal fluid on OCT was achieved during the 1 year follow‐up period. PDT was used, either as monotherapy or in combination with anti‐VEGF injections, in 16 out of these 22 eyes (73%). For non‐PCV MNV, PDT was used in 3 out of 9 eyes (33%) that achieved full resolution of fluid during the 1 year period. In addition to the information provided in Table 2 one eye had also received one intravitreal triamcinolone acetonide injection, one eye had received cataract surgery and one eye had received YAG‐laser‐assisted capsulotomy to treat posterior capsule opacification.

**TABLE 2:** Characteristics at 1 year follow‐up.

	PCV‐associated MNV (39 eyes of 37 patients)	Non‐PCV MNV (18 eyes of 17 patients)	*p*‐value	Total (57 eyes of 53 patients)^c^
During follow‐up period				
Anti‐VEGF treatment	25/39 (64%)	17/18 (94%)		42/57 (74%)
Number of anti‐VEGF injections received – Median [IQR]	6 [5–10]	[5–11]		7 [5–10]
One anti‐VEGF agent used	23/25 (92%)	10/17 (59%)		33/42 (79%)
Two anti‐VEGF agents used	2/25 (8%)	7/17 (41%)		9/42 (21%)
PDT	27/39 (69%)	7/18 (39%)		34/57 (60%)
One PDT treatment	20/27 (74%)	7/7 (100%)		27/34 (79%)
Two PDT treatments	6/27 (22%)	N/A		6/34 (18%)
Three PDT treatments	1/27 (4%)	N/A		1/34 (3%)
Total number of PDT treatments in group	35	7		42
Full‐dose PDT	32/35 (91%)	2/7 (29%)		34/42 (81%)
Half‐dose PDT	3/35 (9%)	5/7 (71%)		8/42 (19%)
Focal laser treatment	6/39 (15%)	N/A		6/57 (11%)
One focal laser treatment	5/6 (83%)	N/A		5/57 (9%)
Two focal laser treatments	1/6 (17%)	N/A		1/57 (2%)
(Sub)retinal haemorrhage	9/39 (23%)	2/18 (11%)	0.287^a^	11/57 (19%)
One subretinal haemorrhage	8/9 (89%)	2/2 (100%)		10/11 (91%)
Two subretinal haemorrhages	1/9 (11%)	N/A		1/11 (9%)
Area of subretinal haemorrhage				
<1× disc area	1/9 (11%)	2/2 (100%)		3/11 (27%)
1–3 disc areas	1/9 (11%)	N/A		1/11 (9%)
> 3 disc areas	1/9 (11%)	N/A		1/11 (9%)
Unknown size	6/9 (67%)	N/A		6/11 (55%)
At 1 year follow‐up				
Visual acuity in logMAR [IQR]—affected eye	0.15 [0.05–0.40]	0.15 [0–0.24]	0.442^b^	0.15 [0.05–0.40]
Visual acuity in logMAR [IQR]—fellow eye	0.05 [0–0.17]	0 [−0.08–0.06]	0.080^b^	0 [−0.03–0.10]
OCT characteristics				
Complete resolution of macular fluid at 1 year follow‐up	16/33 (48%)	5/16 (31%)	0.253^a^	21/49 (43%)
Complete resolution of macular fluid occurred at any time point during the course of 1 year follow‐up	22/32 (69%)	9/16 (56%)	0.393^a^	31/48 (65%)
Treatment that achieved complete resolution of macular fluid				
Bevacizumab monotherapy	4/22 (18%)	2/9 (22%)		6/31 (19%)
Aflibercept monotherapy	N/A	2/9 (22%)		2/31 (6%)
Ranibizumab monotherapy	N/A	2/9 (22%)		2/31 (6%)
PDT monotherapy	2/22 (9%)	1/9 (11%)		3/31 (10%)
Focal laser coagulation monotherapy	1/22 (5%)	N/A		1/31 (3%)
PDT + bevacizumab	11/22 (50%)	2/9 (22%)		13/31 (42%)
PDT + aflibercept	2/22 (9%)	N/A		2/31 (6%)
PDT + focal laser	1/22 (5%)	N/A		1/31 (3%)
Focal laser + bevacizumab	1/22 (5%)	N/A		1/31 (3%)
Maximum anti‐VEGF injection interval in patients who achieved complete resolution of macular fluid—median number of weeks (minimum–maximum)	6 (4–16)	4 (4–16)		5 (4–16)
Subretinal fluid	16/39 (41%)	11/18 (61%)	0.158^a^	27/57 (47%)
Subfoveal	10/16 (63%)	10/11 (91%)		20/27 (74%)
Intraretinal fluid	8/39 (21%)	0/18 (0%)	0.038^a^	8/57 (14%)
Foveal	2/8 (25%)	N/A		2/8 (25%)
Foveal retinal thickness—median [IQR]	127 [90–146]	126 [93–176]	0.707^b^	127 [94–146]
Subfoveal choroidal thickness—median [IQR]	175 [169–233]	262 [195–332]	0.066^b^	211 [169–272]
Subfoveal ELM integrity				
Continuous	27/30 (90%)	10/12 (83%)		37/42 (88%)
Irregular/thinned	1/30 (3%)	0/12 (0%)		1/42 (2%)
Indiscernible	2/30 (7%)	2/12 (17%)		4/42 (10%)
Subfoveal EZ integrity				
Continuous	18/31 (58%)	6/13 (46%)		24/44 (55%)
Irregular/thinned	9/31 (29%)	5/13 (38%)		14/44 (32%)
Indiscernible	4/31 (13%)	2/13 (15%)		6/44 (14%)
Subfoveal RPE integrity				
Continuous	29/32 (91%)	9/13 (69%)		38/45 (84%)
Irregular/thinned	2/32 (6%)	3/13 (23%)		5/45 (11%)
Indiscernible	1/32 (3%)	1/13 (8%)		2/45 (4%)

Abbreviations: anti‐VEGF, anti‐vascular endothelial growth factor; ELM, external limiting membrane; EZ, ellipsoid zone; IQR, interquartile range; logMAR, logarithm of the minimal angle of resolution; MNV, macular neovascularization; N/A, not applicable; OCT, optical coherence tomography; PCV, polypoidal choroidal vasculopathy; PDT, photodynamic therapy; RPE, retinal pigment epithelium.

^a^ Chi‐square test.

^b^ Mann–Whitney *U* test.

^c^ There was one patient who had one eye with a PCV‐associated MNV and 1 eye with a non‐PCV MNV.

## 4 DISCUSSION

In this study, we describe a group of patients with nAMD in one or both eyes, without signs of drusen or other abnormalities such as those from pachychoroid spectrum diseases in either eye on MMI. In this cohort, a relatively large proportion of patients had a polypoidal component in association with the MNV. Although the patients in this cohort did not show drusen, the hallmark sign of AMD (Guymer & Campbell, 2023), the median age of 73 years at presentation with MNV in our cohort does suggest the influence of an age‐related component in the development of these MNVs. However, in our cohort, we did not find a female preponderance, which is a known risk factor for the development of (n)AMD (Klein et al., 1992; Rudnicka et al., 2012). The roughly equal rate of male and female patients in our study could be explained by the high prevalence of PCV within our cohort, which has previously been associated with the male gender (Chaikitmongkol et al., 2020). However, this male preponderance in PCV appears to be most clearly seen in Asian patients, whereas Caucasian patients do show a female preponderance (Corvi et al., 2021). Interestingly, a Japanese study from 2021 by Kamao et al. (2021) has compared two groups of patients with nAMD; one with ‘typical’ drusen and one without ‘typical’ drusen. They found various interesting differences between these groups, namely a younger age at presentation, a higher proportion of PCV and a higher SFCT in the group without ‘typical’ drusen. These findings all seem to be in line with our results in the current study, as they found a mean age of 72 years at presentation, 63% of cases had a PCV‐associated MNV, and the mean SFCT was 260 μm. However, it must be recognized that in their study, the definitions of both absence of drusen as well as presence of pachychoroid disease were much less strict than in our current study. The authors allowed a maximum of 20 medium drusen (63–125 μm in diameter) in the group without ‘typical’ drusen, while more than 20 medium drusen or 1 or more large drusen (>125 μm) were classified as ‘typical’. Furthermore, they describe no MMI analysis of signs of pachychoroid disease other than measuring SFCT. Therefore, the authors suggested that a large part of the group without ‘typical’ drusen may be pachychoroid‐related. In a separate study later performed by Kamao et al. (2024), the authors did make a distinction between patients with ICGA‐based signs of pachychoroid in fellow eyes and found this to be present in 50% of cases. When looking at our own results, we found a slightly higher SFCT in the PCV‐associated group compared to the non‐PCV MNV eyes, which did not reach statistical significance after Bonferroni correction for multiple testing. However, this might be related to the association of PCV with pachychoroid disease, although we did not find other signs of pachychoroid disease on MMI. Interestingly, this difference seems to have disappeared at the 1 year follow‐up moment, with a slight switch toward an opposite trend. This might be the result of the high rate of PDT treatments in the PCV group, which is known to cause a reduction in SFCT after 6–12 months, both in PCV and CSC (Maruko, Iida, Sugano, Furuta, & Sekiryu, 2011; Maruko, Iida, Sugano, Saito, & Sekiryu, 2011).

Visual acuity at baseline was worse in the affected eyes compared to the fellow eyes, and did not differ significantly between PCV‐associated MNV and non‐PCV MNV eyes. At 1 year follow‐up, VA remained stable for the overall group, again with no significant differences between PCV‐associated and non‐PCV MNV groups. In the aforementioned study by Kamao et al. (2021), no differences in VA, central retinal thickness or number of injections were found after 5 years of follow‐up between AMD with and without drusen. In the current study, we did find differences between the group with and without PCV in the treatment strategy received, as PCV‐associated MNV was less frequently treated with monotherapy of intravitreal anti‐VEGF injections and more frequently with combined PDT and anti‐VEGF injections or PDT monotherapy, when compared to non‐PCV MNV. This is likely explained by some studies showing that combination therapy of PDT and anti‐VEGF injections is recommended for PCV (Koh et al., 2017; Lim et al., 2020), although other studies have shown a noninferiority in the response to anti‐VEGF monotherapy when compared to this combination therapy (Chong et al., 2025; Oishi et al., 2013; Ruamviboonsuk et al., 2023, 2025; Wong et al., 2019). In our study, PDT (either monotherapy or combination therapy with anti‐VEGF injections) was used in 73% of PCV‐associated MNV cases that showed a complete resolution during the 1 year follow‐up period, compared to 33% of the eyes with non‐PCV MNV who achieved a complete resolution of macular fluid. However, it should be taken into account that more PCV‐associated MNV cases received PDT than non‐PCV MNV cases (69% vs. 39%). Still, several cases of PCV‐associated MNV in our cohort occurred during the worldwide shortage of verteporfin, the photosensitive medicinal agent required to perform PDT (Sirks et al., 2022, 2024). If this had not been ongoing during the inclusion period of this study, the number of eyes with PCV‐associated MNV that were treated with PDT could have been even higher. Finally, focal laser treatment was used in 15% of PCV‐associated MNV but was not performed in non‐PCV MNV. Studies have shown that focal laser treatment can be an effective treatment for extrafoveal polypoidal lesions (Cheung et al., 2013; Sirks et al., 2023), and this may be especially useful if PDT is not available.

The maximum interval between anti‐VEGF injections that could be reached in eyes that showed complete resolution of macular fluid was slightly higher in PCV‐associated MNV compared to non‐PCV MNV, with a median of 6 weeks versus 4 weeks. We found higher rates of subretinal haemorrhage in PCV‐associated MNV compared to non‐PCV MNV, both at initial presentation (21% vs. 17%) and during the 1 year follow‐up period (23% vs. 11%). Most subretinal haemorrhages were relatively small, and none of these required surgical intervention, which is in line with previous studies that showed that Caucasian patients present with lower rates of submacular haemorrhage and with smaller areas of submacular haemorrhage compared to Asian patients with PCV (Corvi et al., 2021).

The typical natural disease course of AMD starts with small drusen and RPE alterations, progresses to medium and large drusen, and finally progresses to geographic atrophy or nAMD (Guymer & Campbell, 2023). A study by Kim et al. (2022) in a Korean cohort has shown that the cumulative progression of intermediate AMD to nAMD over 5 years is 17%, with older age, the presence of reticular pseudodrusen, and a history of nAMD in the fellow eye as risk factors. A clinical study by Sénéclauze et al. (2024) has shown that the presence of central macular drusen is associated with a fourfold increased risk of developing late AMD, compared to eyes that only had pericentral drusen. However, with the current study, we expand on our previous work (Chung et al., 2017), showing that MNVs can develop without the presence of drusen in either eye. The absence of drusen in the relatively large patient cohort in our current study raises questions about the exact pathogenesis of MNVs in these patients. It is known that AMD is a multifactorial disease, in which both genetic and environmental factors play a role (Guymer & Campbell, 2023). An American twin study from 2005 elucidated that between 56 and 71% of disease severity in AMD can be attributed to genetic aspects (Seddon et al., 2005). Today, over 50 different single‐nucleotide polymorphisms (SNPs) that contribute to the risk of AMD have been discovered, with different effect sizes for different SNPs (den Hollander et al., 2022; Fritsche et al., 2016). While these SNPs associated with drusenoid AMD are related to the complement system (Anderson et al., 2010; Boon et al., 2009; Cipriani et al., 2021), lipid metabolism (Tang et al., 2024), angiogenesis and collagen and extracellular matrix production (Fritsche et al., 2016), these pathways may be less pronounced or different in nAMD without drusen.

Drusen are deposits situated between the RPE and Bruch's membrane, mainly composed of lipoproteins, containing high levels of apolipoprotein E, apolipoprotein A and esterified cholesterol (Curcio, 2018). Other components of drusen include carbohydrates, proteins (such as the aforementioned complement factors) and cellular components such as lipofuscin (Anderson et al., 2010; Hageman et al., 2001). All these particles accumulate partly as a result of physiological excretion by RPE cells, but also have their origin in blood plasma resulting from dietary intake (Bergen et al., 2019; Curcio, 2018). The upregulation of secretory autophagy of RPE cells—resulting in the excretion of cellular components in the extracellular space—has also been demonstrated in histopathological studies (Gurubaran et al., 2025). Subsequent progression to MNV formation is the result of damage to the photoreceptors, RPE, Bruch's membrane and the choriocapillaris (Borrelli et al., 2022; Curcio, 2018; Spaide et al., 2003). Additionally, a malfunction of the choriocapillaris may cause relative ischemia to the overlying Bruch's membrane, RPE and photoreceptors (Spaide et al., 2003). The activation of the complement system leads to the attraction of macrophages, which may cause damage to Bruch's membrane (Schlingemann, 2004). These microscopic breaks in Bruch's membrane may then facilitate the ingrowth of choroidal blood vessels into the sub‐RPE space, causing a type 1 MNV (Borrelli et al., 2022). Considering this cascade leading to MNV formation in drusenoid AMD, it is striking to see MNV formation in our cohort, in eyes that show no drusen on MMI.

In pachychoroid spectrum disease, MNV can also occur in the context of other diseases and pathogenetic mechanisms, such as CSC, or as isolated pachychoroid neovasculopathy (Cheung et al., 2024; Pang & Freund, 2015). The latter is thought to be at least partly due to attenuation and subsequent relative hypoperfusion and ischemia of the choriocapillaris, adjacent to dilated underlying Haller's layers blood vessels (Cheung et al., 2024), in combination with damage to Bruch's membrane and/or the RPE. In the current study, we excluded such patients by carefully reviewing MMI and looking for signs of pachychoroid (e.g. pachyvessels on OCT or ICGA, FISH or DISH on ICGA) and the median SFCT in our patient cohort was 225 μm at baseline, which is within the normal range (Cheung et al., 2024; Pauleikhoff et al., 2024). In summary, we found MNVs in this cohort despite the absence of drusen or signs of other diseases causing MNVs. Given the elderly age of these patients (with a median age of 73 years), we consider these MNVs to likely result from an age‐related process. A potential explanation for the formation of MNV in our patient cohort may be localized predisposing factors, such as localized Bruch's membrane defects and ischemia of unknown origin. Although we excluded patients who reported any previous episode of deterioration of vision, it could be that some have previously had a subclinical episode of CSC or a different disease that could have left subtle scars or damage to the RPE and Bruch's membrane, creating a vulnerability to developing an MNV. Another theory is that there may be subtle precursor lesions to AMD present that are invisible with current MMI techniques. Recently, Chen et al. (2023) have shown that ultrahigh resolution OCT scans are able to show a hyporeflective band between the RPE basal lamina and Bruch's membrane that is not visible on regular standard domain OCT. They hypothesize that in early AMD, this hyporeflective band corresponds to basal laminar deposits (BLamD) (Chen et al., 2023). In a previous work from the group of Curcio, the histopathological presence of BLamD was confirmed to cause a hyporeflective split between the RPE and Bruch's membrane on OCT (Sura et al., 2020). However, they have also shown that in areas where no such split was visible on OCT, a thin layer of BLamD could still be detectable on histopathology images. Considering the fact that this band lies in the same space where deposits accumulate to form drusen, this may implicate a (subclinical) precursor lesion to drusen formation and, therewith, to AMD. It is possible that BLamD was present in our cases and may have played a role in the development of Bruch's membrane fragility and subsequent MNV formation. Interestingly, in one of the largest genome‐wide association studies for AMD, Fritsche et al. (2016) have found specific SNPs that were associated with advanced AMD, but not with intermediate AMD (defined as the presence of pigmentary changes or more than 5 macular drusen >63 μm in diameter). These 10 SNPs were present in 7 different genes (*COL15A1*, *COL8A1*, *MMP9*, *PCOLCE*, *MMP19*, *CTRB1*‐*CTRB*2 and *ITGA7*) that play a role in extracellular matrix composition. In a Chinese cohort of 69 patients, Cao et al. (2019) found a correlation between a SNP in *COL8A1* and what the authors called ‘idiopathic choroidal neovascularization’, defined as the presence of an MNV in a patient younger than 50 years without any macular or retinal disease associated with MNV. Although no studies have explored this, deficits in one or more of these genes related to extracellular matrix composition may lead to reduced stability of Bruch's membrane, making it vulnerable to penetration by choroidal blood vessels, leading to sub‐RPE MNV. Future studies that include additional types of micro‐imaging, such as the aforementioned ultrahigh resolution OCT (Chen et al., 2023), correlation between clinical imaging and histopathology, the use of proteomics and genetic studies that correlate genotyping to the different AMD phenotypes may shed more light on the exact pathogenesis of MNV formation in nAMD without drusen. Future prospective studies with longer follow‐up times can also provide more insight into the prognosis and optimal treatment of nAMD without drusen.

Although the current study provides a relatively large cohort of patients with nAMD without drusen, there are some limitations that are partly due to the retrospective nature of the study. These include the unavailability of all MMI for several patients, the non‐standardized treatment protocol and the lack of 1‐year follow‐up assessment for half of the included eyes. Furthermore, because the majority of patients were referred to us by an ophthalmologist, partly due to a poor response to intravitreal anti‐VEGF injections or due to a suspicion of PCV or another diagnosis, this might cause a selection bias. Finally, including both eyes within the same individual patient might have skewed the data slightly, due to these eyes being related to one another.

In conclusion, we describe a cohort of patients with nAMD without drusen. We show that drusen are not mandatory in some patients to develop MNV at an older age, also when there are no signs of other underlying diseases. This indicates that nAMD comprises a broad phenotypic spectrum that not only suggests differences in pathogenesis but may also have important implications for treatment and visual prognosis. Using extensive and detailed MMI, as well as histopathological, genetic and metabolomic studies, future studies may further dissect the aetiology and clinical outcome of these subtypes of neovascular maculopathy in the elderly population.

## CONFLICT OF INTEREST STATEMENT

MJS, EHCvD, HG, SB, WY, SY, JM, ROS, RMHD and CJFB report no possible conflicts of interest with regard to this study.
